# Network Fluctuations Hinder Cooperation in Evolutionary Games

**DOI:** 10.1371/journal.pone.0025555

**Published:** 2011-10-19

**Authors:** Alberto Antonioni, Marco Tomassini

**Affiliations:** Information Systems Department, Faculty of Business and Economics, University of Lausanne, Lausanne, Switzerland; University of Zaragoza, Spain

## Abstract

In this paper we study the influence of random network fluctuations on the behavior of evolutionary games on Barabási–Albert networks. This network class has been shown to promote cooperation on social dilemmas such as the Prisoner's Dilemma and the Snowdrift games when the population network is fixed. Here we introduce exogenous random fluctuations of the network links through several noise models, and we investigate the evolutionary dynamics comparing them with the known static network case. The results we obtain show that even a moderate amount of random noise on the network links causes a significant loss of cooperation, to the point that cooperation vanishes altogether in the Prisoner's Dilemma when the noise rate is the same as the agents' strategy revision rate. The results appear to be robust since they are essentially the same whatever the type of the exogenous noise. Besides, it turns out that random network noise is more important than strategy noise in suppressing cooperation. Thus, even in the more favorable situation of accumulated payoff in which links have no cost, the mere presence of random external network fluctuations act as a powerful limitation to the attainment of high levels of cooperation.

## Introduction

In the last decade, thanks to advances in network science, a large number of studies dealing with evolutionary games on networks have appeared. The underlying idea is that the classical infinite, well-mixed populations used in the theory [1] are not a particularly good approximation to the actual shape of the contacts that take place in society, although they do allow rigorous mathematical results to be reached. Indeed, social interactions between agents are mediated by finite networks of contacts, which is why there is a strong interest in the study of this kind of collective systems. For a synthesis of the main results obtained in the last years, we refer the reader to the following reviews which, altogether, give the state of the art in the field of evolutionary games on networks [2]–[4].

At the beginning, investigations were targeted at static networks, i.e. networks that do not change during time. A very good summary of this case is provided by Roca et al. [3]. This is an acceptable approximation when network changes are slow with respect to behavioral changes of the agents and it is a useful first step. However, actual social networks are dynamical entities in which agents may leave the network, new agents may join it, and links can be formed and dismissed as well. So, the more general models should be dynamical, and several approaches have been suggested to deal with these time-dependent aspects of the network structure in evolutionary games; an excellent recent review is contained in [4]. Most models assume a constant population structure, i.e. no agent leaves or join the network, which means that the system is at equilibrium with respect to exchanges of “matter”. This of course removes the need of dealing with the rate of change 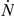 of *N*, since 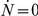. The condition also holds when the number of agents entering the system is the same as those leaving it, but this would complicate matters since contacts would change. For this reason it is assumed that there is no flow through the system boundaries. On the other hand, the number of links *L* may be subject to internal change and, even if *L* stays constant (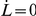), it will generally be the case that links are actually being rewired among different pairs of agents. The above is the most often used scenario, although some works have also dealt with growing networks under strategic conditions, e.g. see [5] and the abundant economic literature on strategic network formation as summarized, for example, in [6]. In all cases, only pairwise interactions are considered at first: although *n*-person interactions are important, it is believed that two-person games are a first useful and necessary step and will be assumed here too.

Now, link rewiring can be either an exogenous random phenomenon, or it can obey some other rule. If it is completely random, then the network drifts toward randomness itself, in the sense that its degree distribution tends to be Poissonian. This is not an interesting case since we already know from the static case [3], [7] that random graphs are not particularly conducive to cooperative interactions between agents. Besides, actual social networks are not random and thus this is not a realistic case either. Thus others, perhaps more socially-inspired relinking patterns, have been postulated. For example, the models presented in [8], [9] try to take into account a kind of strategic “negotiation” between the concerned pair of connected nodes in order to decide whether a given link must be cut or not, while in other cases the decision is unilateral [10], [11] and only affects certain types of links, usually defector-defector ones. The rewiring phase, on the other hand, has often been related to triadic closure i.e., the formation of links among agents that have a neighbor in common have been favored [9], [10]. Starting from a random graph whose clustering coefficient tends to 0 as 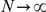, this will cause a bias towards an increase of the mean clustering coefficient of the whole network. The reason behind this bias is that triadic closure is a well known observed feature of actual social networks. A recent related theoretical work on the dynamics of link rewiring in games, using explicit rewiring probabilities based on link type and Markov chains analysis has appeared in [12].

In this study we take a different approach and treat network dynamics as an *exogenous* phenomenon that is undoubtedly present to a larger or smaller extent but of which we do not know neither the exact origins nor the actual stochastic behavior. Thus, we shall assume that the network links are simply subject to noise. This point of view is justified by the fact that there is a large variety of social networks and, although some global statistical features such as degree distribution, mean degree, degree correlations, mean clustering coefficient and so on tend to be similar across networks, there is not, as yet, a general theory that explains every single network aspect when it comes to their dynamical behavior. In other words, instead of formulating some mechanisms that are supposed to be responsible for link evolution, we shall take inspiration from empirical data coming from some time-resolved studies of social network evolution to postulate general forms of network noise that are expected to describe, in a statistical sense, how the network links fluctuate. It is not the case that strategic network formation issues are unimportant; we only think that many networks are under the influence of largely unknown exogenous dynamically changing variables and we would like to lump all of them together under the form of unspecified fluctuations. The following step is to study through numerical simulations the effects of such network fluctuations on the behavior of paradigmatic evolutionary games.

### Games on Networks

We have studied the four classical two-person, two-strategies games described by the payoff bi-matrix of Table 1.

**Table 1:** Generic payoff bi-matrix for the two-person, two-strategies symmetric games.

	*C*	*D*
*C*	(*R*,*R*)	(*S*,*T*)
*D*	(*T*,*S*)	(*P*,*P*)

*C* and *D* are the possible strategies, and *R,T,P*, and *S* are payoff values as discussed in the text.

In this matrix, *R* stands for the *reward* the two players receive if they both cooperate (*C*), *P* is the *punishment* for bilateral defection (*D*), and *T* is the *temptation*, i.e. the payoff that a player receives if she defects while the other cooperates. In the latter case, the cooperator gets the *sucker*'*s payoff S*. The parameters' values are restricted to the standard configuration space defined by *R* = 1, *P* = 0, −1≤*S*≤1, and 0≤*T*≤2. In the resulting *TS*-plane, each game's space corresponds to a different quadrant depending on the ordering of the payoffs. If the payoff values are ordered such that *T*>*R*>*P*>*S* then defection is always the best rational individual choice, so that (*D,D*) is the unique Nash Equilibrium (NE) and also the only Evolutionarily Stable Strategy (ESS) [1] and we get the *Prisoner*'*s Dilemma* (PD) game. Mutual cooperation would be socially preferable but *C* is strongly dominated by *D*.

In the *Snowdrift* (SD) game, the order of *P* and *S* is reversed, yielding *T*>*R*>*S*>*P*. Thus, in the SD when both players defect they each get the lowest payoff. (*C,D*)and (*D,C*) are NE of the game in pure strategies. There is a third equilibrium in mixed strategies which is the only dynamically stable state, while the two pure NE are not [1]. Players have a strong incentive to play *D*, which is harmful for both parties if the outcome produced happens to be (*D,D*).

With the ordering *R>T>P>S* we get the *Stag Hunt* (SH) game in which mutual cooperation (C,*C*) is the best outcome, Pareto-superior, and a NE. The second NE, where both players defect is less efficient but also less risky. The dilemma is represented by the fact that the socially preferable coordinated equilibrium (C,*C*) might be missed for “fear” that the other player will play *D* instead. The third mixed-strategy NE in the game is evolutionarily unstable [1].

Finally, the *Harmony* game has *R>S>T>P* or *R>T>S>P*. *C* strongly dominates *D* and the trivial unique NE is (C,*C*). This game is non-conflictual by definition and does not cause any dilemma: we include it just to complete the quadrants of the parameter space.

With the above conventions, in the figures that follow, the PD space is the lower right quadrant; the SH is the lower left quadrant, and the SD is in the upper right one. Finally, Harmony is represented by the upper left quadrant.

## Results

Recent research on evolutionary games on static networks has shown that network reciprocity effects may favor cooperation to a fair extent in games, such as the PD, in which it would be doomed if the interacting population were well mixed [3], [7], [13]. In particular, largely degree-inhomogeneous networks topologies such as Barabási–Albert (BA) scale-free networks seem to possess the ingredients that boost cooperation the most. Network reciprocity in this case is facilitated and stabilized by cooperators that get hold of hub nodes, are surrounded mostly by cooperators, and are connected to other cooperator hubs [14]. Social networks also seem to be able to enhance cooperation [15], [16], albeit to a lesser extent than the ideal scale-free case. In social networks too there is degree inhomogeneity expressed by broad-scale degree distribution functions, although usually the tails fall off faster than in scale-free networks. Here other mechanisms play a role besides highly connected nodes: they manifest themselves through clustering and the presence of community boundaries, which are almost absent in BA networks. These features of actual social networks may favor cooperation with respect to well mixed populations. Because they are the best cooperation amplifiers among the studied network models, and thus they represent a kind of upper bound, we focus our numerical simulation study on Barabási–Albert scale-free networks. The construction of BA networks is well known and will be briefly described in the Methods section. The simulations start by randomly distributing cooperators and defectors among the networks' nodes in the same proportion. The simulations then proceed until a steady state is reached and, at this point averages are computed. In a steady state strategy fluctuations are smoothed out both in static and noisy networks. For more details the reader is referred to the Methods section.

### Sequence of Random BA networks

The first numerical experiment is to compare the behavior of evolutionary games on static BA networks and time-varying networks of the same family. In the latter case, we create a stochastic process 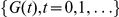 in which each *G*(*t*) is an independently generated BA graph with the same size and mean degree while in the static case there is a single graph *G*(0) which is used all along. Clearly, by construction all the graphs in the sequence 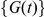 have equivalent degree distributions. During an epoch *t* the players, which initially randomly receive a strategy 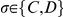, will synchronously play the given game with their neighbors. In the dynamic case, with a certain frequency 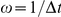, the population graph is rebuilt at each 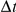 time steps. Players are numbered, and their current strategies are conserved when the network changes, but their neighborhood will in general be different. This process is not a likely one socially because it entails too much uncorrelated change, but it is simple and clear from a theoretical point of view. It will thus be used as a benchmark case in the following, as it represents the extreme case in which there is no correlation between successive instances of the network and each new network is an i.i.d. random variable. In all cases we start with the same number of cooperators and defectors randomly distributed over the network nodes. Other initial proportions are also interesting to investigate, as it has been done for the static case by Roca et al. [3]. Here, however, we shall focus on the comparison between the static and the dynamic cases and not so much on the robustness of results with respect to the initial conditions.

**Figure 1:**
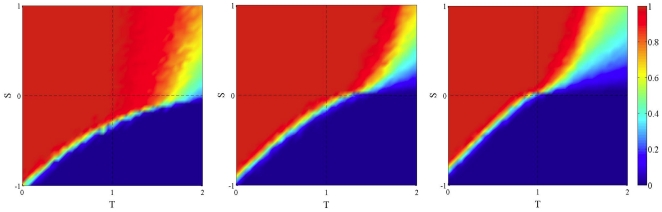
Asymptotic distribution of strategies in the TS plane in static and dynamic BA networks using replicator dynamics as an update rule. Initial density of cooperators is 0.5 uniformly distributed at random in all cases. Leftmost image: the static case. Middle image: frequency 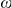 of network generation is 0.1; rightmost image: 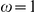. Values are averages over 100 independent runs.

Fig. 1 shows the average amount of cooperation at the end of the simulations on dynamically generated BA networks (central and right image) with respect to the static case (leftmost image). The strategy update rule is replicator dynamics (see Methods Sect. for details on this revision protocol).

The trend is clear: cooperation is negatively affected by the network noise, and the effect is more pronounced the higher the noise from left to right. The two games that are the most affected are the PD and the SH, while the SD game is the least affected. This was expected since, while PD and SH have monomorphic dynamically stable states, in the SD the equilibrium state is a dimorphic population.

**Figure 2:**
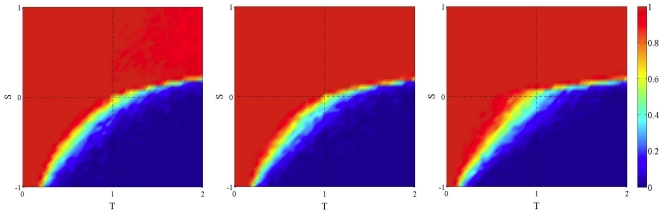
Asymptotic distribution of strategies in the TS plane in static and dynamic BA networks using unconditional imitation of the best neighbor as an update rule. Initial density of cooperators is 0.5 uniformly distributed at random in all cases. Leftmost image: the static case. Increasing towards the right: frequency 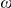 of network generation is 0.1 and 1. Values are averages over 100 independent runs.

Figure 2 shows the same dynamics but using unconditional imitation of the best instead of replicator dynamics. Here the focal player imitates the strategy of the neighbor having obtained the highest payoff, including himself (see Methods section). Looking at the static case (leftmost image) it is already clear that there is a lower amount of cooperation to start with in the PD quadrant, as well as in the SH case with respect to random graphs, while cooperation is high for the SD game (see Roca et al. [3] for a detailed discussion of these effects). Adding network noise has little effect but still the tiny amount of cooperation existing is almost completely lost when the noise level reaches the value one. A tentative qualitative explanation of the relative insensitivity to noise in this case is the following. The way in which a new network is generated in the noisy case (see above) tells us that, on the average, a given player will have more or less the same proportion of cooperators and defectors as neighbors in the new network as in the previous one. Since deterministic unconditional imitation rule depends on the global state of the neighborhood, it seems likely that the network dynamics will not have a large effect in this case.

Table 2 summarizes the numerical results by giving the average value of cooperation in the three non-trivial games for static and dynamic networks, and for the two strategy revision rules. From the table, the trend towards loss of cooperation in randomly fluctuating networks becomes very clear.

**Table 2:** Asymptotic average cooperation fraction in static and noisy BA networks.

	PD, ib	PD, rd	SD, ib	SD, rd	SH, ib	SH, rd
Static BA networks	0.030	0.131	0.863	0.823	0.597	0.615
Dynamic Network ( 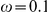 )	0.027	0.025	0.890	0.778	0.582	0.490
Dynamic Network ( 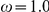 )	0.021	0.009	0.870	0.572	0.538	0.364

‘ib’ and ‘rd’ stand for ‘imitate the best’ and ‘replicator dynamics’ update rules respectively. PD, SD, and SH design the Prisoners Dilemma, Snowdrift, and Stag Hunt games respectively. The table refers to Figs. 1 and 2.

### Fluctuations from Network Edge Swap

**Figure 3:**
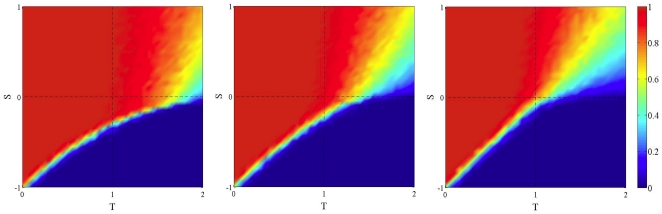
Asymptotic distribution of strategies in the TS plane in static and dynamic BA networks using replicator dynamics as an update rule. Strategy update dynamics is asynchronous and the initial BA graph is rewired as explained in the text. Initial density of cooperators is 0.5 uniformly distributed at random in all cases. Leftmost image: the static case. Middle image: frequency 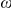 of network rewiring is 0.1; rightmost image: 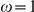. Values are averages over 100 independent runs.

The independent sequence of BA graphs used above constitutes an ideal situation that can be considered as a baseline case, but it is quite far from a realistic link evolution in a given single network. To do a step towards more realism, we shall now assume that the sequence of graphs 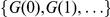 is generated by successively rewiring an initial given graph as suggested in [17]. We begin with *G*(0) being a Barabási–Albert network; then, starting at time *t* = 1, each successive graph *G*(*t*) in the sequence is generated by swapping two randomly chosen non-adjacent pair of edges in the previous graph *G*(*t*−1). In contrast to the previous case, where the sequence of graphs was an i.i.d. one, this process is a Markov chain since each new graph in the sequence depends on the previous one. The edge swap preserves the degree distribution *P*(*k*) of *G* and obviously the node's degree and the mean degree as well. The graphs, however, become more and more randomized as time goes by, as they tend to loose the historical degree correlations between hubs that arise in the original BA construction. Note that in this case we assume an asynchronous dynamics since it is, in our opinion, qualitatively more adapted to the new situation. Results are almost the same with either synchronous or asynchronous dynamics as shown in [3]. For the BA networks and replicator dynamics, this is also clear from the leftmost images in Figs. 1 and 3. Thus, instead of updating all the players' strategies at once in each time step as before, we randomly choose a player to update (with replacement). This is called an elementary time step. The period of network rewiring in this case is the number of elementary steps before an edge swap takes place, and the frequency *ω* is just the reciprocal of this number.

The visual results for this kind of generic network noise under replicator dynamics are shown in Fig. 3, while the measured final average values are given in Table 3. It is clear that, in agreement with the previous model of noise, even a moderate amount of network links fluctuation gives rise to a serious loss of cooperation in all the non-trivial games. To be more precise, after a good deal of edge swapping, the resulting networks, although they keep the original degree distribution, are close to scale-free random graphs generated according to the configuration model [17]. Cooperation frequencies on random scale-free graphs of the latter type are still better than random Erdös-Rényi graphs results (see [8] and especially [18], where a complete analysis of cooperation in the PD in random scale-free graphs is provided). However, they are significantly lower than those found in BA networks due to the loss of some early hubs interconnections that are present in BA networks because of the temporal growing process [18]. Thus, the reasons for the loss of cooperation are both the topology changes induced by the above loss of interconnected hubs, and especially the noisy neighborhoods induced by the edge swaps.

**Table 3:** Asymptotic average cooperation fraction in static and dynamic networks with edge swap using replicator dynamics as a strategy update rule.

	PD	SD	SH
Static BA Networks	0.131	0.825	0.617
Dynamic Network ( 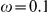 )	0.055	0.699	0.576
Dynamic Network ( 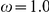 )	0.017	0.591	0.523

Values refer to Fig. 3.

### Fluctuations from Edge Rewiring

Once more, the previous assumed network fluctuation, although it is of theoretical interest, is still far from what happens in real networks. Experimental observations on dynamically changing social networks show that global statistics such as *P*(*k*), the mean degree 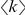, the mean clustering coefficient 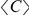 all remain similar but not exactly the same, they fluctuate to some extent during network evolution. This is true for growing networks, which are the majority of those that have been observed, but also for time-resolved studies of constant-size ones. These kind of results have been reported, among others, in [19]–[23]. Inspired by these considerations, we shall thus examine a third random dynamics that, without making strong assumptions on how players have their links cut and rewired, nevertheless provides fluctuations of the main network quantities similar to what seems to happen in real social networks. This should allow us to check whether the conclusions reached with the two previous models are robust enough starting from a BA network. Rewiring works as follow:

1. a node *i* is chosen with probability proportional to its degree *k_i_* and one of its neighbors 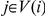 is selected with uniform probability
2. the corresponding 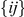 link is suppressed
3. node *j* creates a new link with a node 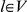 anywhere in the graph with probability proportional to *l*'s degree (preferential attachment)
4. to conserve minimum degree *k_min_*, if nodes *i* or *j* have degree *k_min_* they are not considered for rewiring and two other nodes are selected

**Figure 4:**
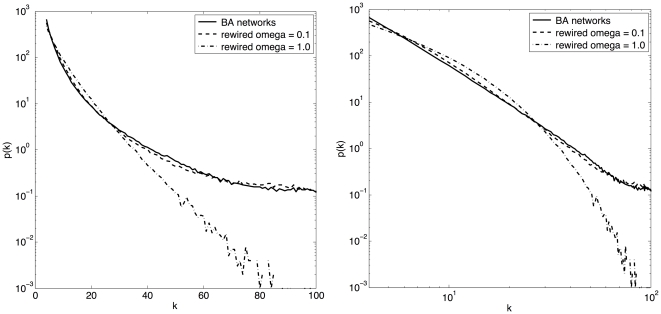
Empirical degree distribution functions for the original BA networks and for the final rewired ones. Left image: lin-log scales; right image: log-log scales. The distributions for the rewired graphs are shown for two levels of network noise. For high levels of noise, distributions tend to the exponential type, otherwise they are closer to the original power-law. Values are averages over 1000 graph realizations for each curve.

This process makes highly connected nodes more likely to loose a link but, on the other hand, it also gives them more probability of being chosen for a new connection. The network statistics do change but they remain relatively close to the starting BA graph. In our simulations, after many rewirings, the network degree distribution function does remain broad-scale in average, but the tail tends to fall off faster than the original power-law. Figure 4 shows the degree distribution functions for the original and the rewired networks for two levels of noise averaged over 1000 graph realizations. For the rewired networks, the graphs refer to the final configurations. From the curves, one can see that for low noise (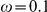) the rewired networks have almost the same distribution as the original BA ones. On the other hand, when the noise is high (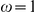) the networks undergo a more marked change and the resulting degree distributions are closer to an exponential, as seen in the left image of Fig. 4 where the scales on the axes are lin-log. Indeed, most empirical degree distributions sampled on actual static social networks do give results that are between these two limit cases, i.e. a power-law and an exponential distribution [24], [25].

**Figure 5:**
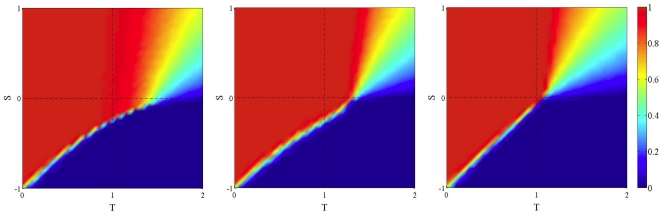
Asymptotic distribution of strategies in the TS plane in rewired networks using replicator dynamics. Strategy update dynamics is asynchronous. Leftmost image: static rewired network (see text). Middle and rightmost images refer to dynamic graphs with frequency 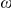 of network rewiring of 0.1 and 1, respectively. Initial density of cooperators is 0.5 uniformly distributed at random in all cases. Values are averages over 100 independent runs.

The simulations proceed in an asynchronous manner, as explained in the edge swap case above. Figure 5 depicts the behavior of the four game classes on networks undergoing the above link fluctuations. The leftmost image is given for comparison; it refers to a static graph that has been first rewired so as to randomize the links as explained above, before being used as a fixed population topology. The middle and rightmost images depict the noisy cases with a frequency of rewiring of 0.1 (middle) and 1 (right). It is to be remarked that the static rewired network does not become an Erdös-Rényi random graph, and still allows for a fair amount of cooperation with respect to the pure BA case reported in the leftmost image of Fig. 3. Thus, the loss of cooperation observed as the network fluctuates is significant and can lead to full defection for the PD when rewiring and revising strategy have the same time scale (rightmost image). Snowdrift and SH are less negatively affected. The average final values for the three games are reported in Table 4. The conclusion that we can draw from the results obtained with the three network fluctuation models is the following: whatever the source of random link fluctuations, as soon as the amount of noise becomes non-negligible i.e., as soon as network changes are at least ten times slower than strategy revision, the cooperation levels observed on static networks become weaker and they are completely lost when link noise and strategy update occur at the same rate. This conclusion is valid for all the non-trivial games studied, but it is particularly visible in the PD quadrant where defection becomes complete for 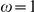.

**Table 4:** Asymptotic average cooperation fraction in static and dynamic networks with edge rewiring (see text) using replicator dynamics as a strategy update rule.

	PD	SD	SH
Static Networks	0.072	0.696	0.588
Dynamic Network ( 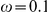 )	0.057	0.678	0.595
Dynamic Network ( 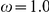 )	0.009	0.544	0.528

Values refer to Fig. 5.

### Network and Strategy Noise

**Figure 6:**
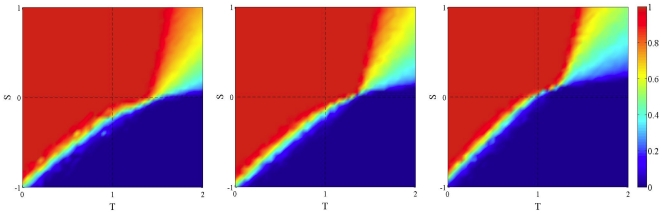
Asymptotic distribution of strategies in the TS plane in static and dynamic BA networks using the Fermi rule (see text). Initial density of cooperators is 0.5 uniformly distributed at random in all cases. In all cases *β* = 0.1. Leftmost image: the static case. Middle image: frequency of graph renewal 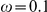. Right image: 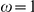. Values are averages over 100 independent runs.

Until now, we have studied the impact of network fluctuations on typical evolutionary games. Another common source of noise in games arises from strategy errors. These are meant to capture various sources of uncertainty such as deliberate and involuntary decision errors which might play the role of experimentation in the environment, or be related to insufficient familiarity with the game. One easy way to include strategy noise is to use the Fermi function [2] as an update rule (see the Methods section for definitions). The parameter *β* in the function gives the amount of noise: a low *β* corresponds to high probability of error and, conversely, high *β* means that errors will be rare. One may ask how much these errors influence cooperation in networks of contacts, and whether they combine positively or negatively with network noise. As for their influence on static BA networks, the answer has been given in [3], where it is shown that for low noise (*β = *10) the equilibrium behavior is similar to the one seen with replicator dynamics, while values of *β* close to 0.01 are enough to suppress all residual cooperation in the PD. In this case selection is weak, payoffs and network structure play a less important role. In other words, only comparatively high rates of strategy errors are really detrimental to cooperation. But when network fluctuations are present, cooperation is quickly lost, even for values of *β* that still allow for a fair amount of cooperation in the static case. Figure 6 shows this for a static network (leftmost image) as well as for two levels of network noise (central and right image) for *β* = 0.1. Network noise has been created as in our first model, i.e. by generating a sequence of independent BA networks with frequency 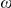.

Table 5 gives the asymptotic average values of cooperation in the three non-trivial games for static and dynamic networks. Although in the static case there is still a certain amount of cooperation in spite of the fact that *β* is relatively low, adding network noise quickly makes the situation worse. In conclusion, we can say that both kinds of errors tend to hinder cooperation, but network noise is more important than strategy noise in disrupting cooperation on degree-heterogeneous networks.

**Table 5:** Asymptotic average cooperation fraction in static and dynamic BA networks using the Fermi rule with *β = *0.1 (see text).

	PD	SD	SH
Static BA networks	0.066	0.817	0.574
Dynamic Network ( 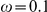 )	0.035	0.739	0.552
Dynamic Network ( 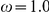 )	0.001	0.565	0.416

Values refer to Fig. 6.

## Discussion

The finding that fixed Barabási–Albert scale-free networks of contacts notably increase cooperation in social dilemmas has been an important one [8], [14] and has raised substantial hope, since scale-free graphs are better representations of actual networks of contacts than the random graphs and regular lattices that have often been used in the past. However, subsequent studies have somehow reduced its scope for various reasons. In the first place, the gains in cooperation can be partially or totally offset if players must pay an extra cost in order to maintain more contacts, as suggested by Masuda [26]. In a similar way if average, instead of accumulated payoff is used, the advantage of degree heterogeneity is lost as the individual's payoff is divided by its degree in the graph [27], [28]. The latter seems to be an extreme case but it still shows in another way that the cost for an agent to maintain few or many links cannot be the same, although it certainly depends on the social context. Furthermore, if the players' decision rule is partially conventional, some of the advantage is equally lost. For example, this has been shown to happen when agents have a conformist component to their behavior [29]. Finally, even when none of the above applies, the amount of cooperation gain due to network reciprocity can still be slim or non-existent depending on the strategy update rule and several other factors. This has been shown, among many other things, in the extensive studies of Roca et al. [3], [30] where it appears that using deterministic best response as an update rule instead of an imitative rule such as replicator dynamics causes a serious loss of cooperation in the PD on BA scale-free networks which recover the mean-field case.

In the present work, inspired by the empirical observation that networks are never completely static, we have shown that several forms of random fluctuation of the network links lead to a marked loss of cooperation that affects all the games' phase space, even for moderate amounts of noise. The result is robust because, irrespective of the precise form of network noise, the same phenomenon manifests itself: asymptotically cooperation tends to disappear in the PD, and it diminishes in the other games. Moreover, network fluctuations appear to be more important than strategy noise in provoking a loss of cooperation. All the above refers to BA scale-free graphs and the general conclusion is that these population structures are not robust enough as cooperation amplifiers, as many factors may contribute to impair the ideal results. Network fluctuations, which certainly must occur in real-life, are among the most important factors. As a result, it can be said that, when the amount of noise is non-negligible, the system tends to behave in a mean-field way and thus the well-mixed population description seems to be adequate. This can be seen visually by comparing our figures with 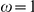 with those for complete graphs that appear in [7] and [3]. The main reason for this behavior is the fluctuation of the neighborhood seen by each agent due to global network noise which, to some extent, resembles population mixing.

However, it has to be said that these model networks, although similar in some sense, do not represent well enough actual social networks; for instance, they do not have enough clustering, community structure, and degree correlations, among others. It would be interesting to see what is the effect of noise on games on social networks. Work is in progress in this direction. Finally, the negative conclusion that cooperation in scale-free networks is hindered by exogenous random network dynamics, should be taken with caution. It is valid when strategy evolution and network dynamics are completely uncorrelated as it was the case in the present study. However, it has been shown that when cutting and forming links in a co-evolving network has a strategic dimension to it, then cooperation can thrive and be stable since severing and reforming links is purposeful and based either on game payoff, or on game-related considerations (see, for instance, [4], [8]–[10]). From a social point of view, the difference is whether an agent can purposefully manipulate her environment, or is just under the influence of external network forces that she cannot control. In our opinion, both cases, as well as mixed situations may exist in reality. The study presented here belongs to the first stylized situation.

## Methods

### Population Structure

The population of players is a connected undirected graph *G*(*V,E*), where the set of vertices *V* represents the agents, while the set of edges *E* represents their symmetric interactions. The population size *N* is the cardinality of *V*. The set of neighbors of an agent *i* is defined as: 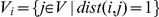, and its cardinality is the degree 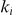 of vertex 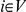. The average degree of the network is called 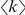 and 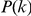 denotes its degree distribution function, i.e. the probability that an arbitrarily chosen node has degree *k*. For the network topology we use the classical Barabási–Albert [31] networks. BA networks are grown incrementally starting with a clique of *m*_0_ nodes. At each successive time step a new node is added such that its *m*≤*m*_0_ edges link it to *m* nodes already present in the graph. It is assumed that the probability *p* that a new node will be connected to node *i* depends on the current degree *k_i_* of the latter. This is called the *preferential attachment* rule. The probability 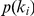 of node *i* to be chosen is given by 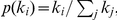 where the sum is over all nodes already in the graph. The model evolves into a stationary network with power-law probability distribution for the vertex degree 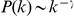, with 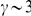. For the simulations, we started with a clique of *m*_0_ = 9 nodes and, at each time step, the new incoming node has m = 4 links.

### Payoff Calculation and Strategy Revision Rules

In evolutionary game theory, one must specify how individual's payoffs are computed and how agents decide to revise their present strategy. In the standard theory, there is a very large well-mixed population; however, when the model is applied to a finite population whose members are the vertices of a graph, each agent *j* can only interact with agents contained in the neighborhood *V*(*j*), i.e. only local interactions are permitted.

Let 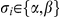 be the current strategy of player *i* and let us call *M* the payoff matrix of the game. The quantity
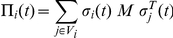



is the accumulated payoff collected by agent *i* at time step *t* and 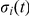 is a vector giving the strategy profile at time *t*. Several strategy update rules are commonly used. Here we shall describe three of them that have been used in our simulations.

The first rule is to switch to the strategy of the neighbor that has scored best in the last time step. This *imitation of the best* policy can be described in the following way: the strategy 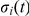 of individual *i* at time step *t* will be
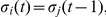



where
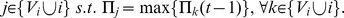



That is, individual *i* will adopt the strategy of the player with the highest payoff among its neighbors including itself. If there is a tie, the winner individual is chosen uniformly at random, but otherwise the rule is deterministic.

The *local replicator dynamics* rule is stochastic [32]. Player *i*'s strategy 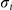 is updated by drawing another player *j* from the neighborhood *V_i_* with uniform probability, and replacing 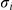 by 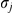 with probability:
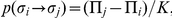



if 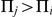, and keeping the same strategy if 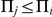. 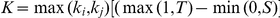, with *k_i_* and *k_j_* being the degrees of nodes *i* and *j* respectively, ensures proper normalization of the probability 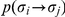.

The last strategy revision rule is the *Fermi rule* [2]:
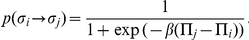



This gives the probability that player *i* switches from strategy 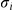 to 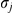, where *j* is a randomly chosen neighbor of *i*. 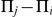 is the difference of payoffs earned by *j* and *i* respectively. The parameter *β* in the function gives the amount of noise: a low *β* corresponds to high probability of error and, conversely, high *β* means low error rates. This interpretation comes from physics, where the reciprocal of *β* is called the temperature. Consequently, payoffs will be more noisy as temperature is raised (*β* is lowered).

### Simulation Parameters

The BA networks used in all simulations are of size *N* = 2000 with mean degree 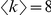. The *TS* plane has been sampled with a grid step of 0.05 and each value in the phase space reported in the figures is the average of 100 independent runs, using a fresh graph realization for each run. The initial graph for each run doesn't change in the static case, while it evolves in the dynamic case, as described in the main text. Note that steady states have always been reached when strategies evolve on a static graph. We first let the system evolve for a transient period of 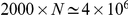 time steps. After a steady state is reached past the transient, averages are calculated during 200×*N* additional time steps. True equilibrium states in the sense of stochastic stability are not guaranteed to be reached by the simulated dynamics. For this reason we prefer to use the terms steady states which are states that have little or no fluctuation over an extended period of time. In the case of fluctuating networks, the system as a whole never reaches a steady state in the sense specified above. This is due to the fact that the link dynamics remains always active. However, the distribution of strategies on the network does converge to a state that shows little fluctuation, i.e. a steady state.
